# Application of machine learning for prognostic modeling in unresectable pancreatic cancer treated with chemoradiotherapy

**DOI:** 10.3389/fonc.2025.1644141

**Published:** 2025-09-01

**Authors:** Wei Xiao, Binbin Yang, Shanbao Ke

**Affiliations:** ^1^ Department of Gastroenterology, Henan Provincial People’s Hospital, Zhengzhou University People’s Hospital, Zhengzhou, Henan, China; ^2^ Division of Gastroenterology, Union Hospital, Tongji Medical College, Huazhong University of Science and Technology, Wuhan, Hubei, China; ^3^ Department of Oncology, Henan Provincial People’s Hospital, Zhengzhou University People’s Hospital, Zhengzhou, China

**Keywords:** artificial intelligence, survival, pancreatic cancer, chemoradiotherapy, model

## Abstract

**Background:**

Patients with unresectable pancreatic cancer have poor outcomes despite chemoradiotherapy (CRT). Traditional prognostic tools lack accuracy in predicting survival. This study aimed to develop an artificial intelligence (AI)-based model to improve survival prediction.

**Methods:**

We retrospectively included 214 patients treated with CRT between 2018 and 2024. Five models—Cox, LASSO, RSF, SVM, and XGBoost—were trained to predict overall survival. Model performance was evaluated using the C-index, time-dependent ROC, calibration, and decision curve analysis. SHAP was used to interpret feature importance.

**Results:**

The median overall survival (mOS) for the entire cohort was 18.4 months (95% CI, 16.3–28.1). XGBoost showed the best performance (C-index = 0.949). It also achieved higher area under the receiver operating characteristic curves at 6 and 12 months (0.751 and 0.732) compared to other models. Calibration and clinical benefit were superior. SHAP analysis identified CA199, tumor size, platelet count, and age as the most important predictors. The model stratified patients into risk groups with significant survival differences (p < 0.001).

**Conclusion:**

The XGBoost-based model accurately predicted survival in unresectable pancreatic cancer patients receiving CRT. It may serve as a useful tool for personalized risk assessment and treatment planning.

## Background

Pancreatic cancer ranks among the most lethal malignancies globally, with a five-year survival rate below 10% (1). It is the seventh leading cause of cancer-related death worldwide and continues to rise in incidence, particularly in developed countries. The disease is typically asymptomatic in early stages, often leading to diagnosis at an advanced or metastatic stage (2). Due to its aggressive biological nature and lack of effective screening tools, most patients present with inoperable tumors at the time of diagnosis (3).

For patients with unresectable pancreatic cancer, concurrent chemoradiotherapy (CRT) is a widely used treatment modality (4). CRT may offer disease control, symptom relief, and modest survival benefits in patients who are not candidates for surgery (5, 6). However, responses to CRT vary substantially among individuals, and survival outcomes remain poor overall. Traditional prognostic tools based on clinical stage, tumor burden, and serum biomarkers provide limited precision in forecasting treatment response or long-term survival in this population (7).

The emergence of artificial intelligence (AI) and machine learning has introduced new opportunities in the field of oncology. These methods can incorporate complex, high-dimensional data to reveal non-linear interactions and latent patterns not captured by conventional statistical techniques (8, 9). AI-based survival models, including Cox regression, LASSO, random survival forests (RSF), support vector machines (SVM), and eXtreme Gradient Boosting (XGBoost), have shown superior performance in various cancer types by improving prediction accuracy and enabling personalized risk estimation (10–13).

This study aimed to apply and compare five AI-assisted modeling approaches—Cox, LASSO, RSF, SVM, and XGBoost—to develop a reliable prognostic model for patients with unresectable pancreatic cancer undergoing CRT. By identifying an optimal prediction framework, we hope to provide a clinically applicable tool for risk stratification, treatment guidance, and individualized patient management in advanced pancreatic cancer.

## Methods

### Patients

This retrospective study included patients diagnosed with unresectable pancreatic cancer from three tertiary hospitals between January 2018 and December 2024. All patients CRT as part of their initial treatment. Clinical, laboratory, and imaging data were collected from institutional databases. Duplicate cases and records with incomplete survival data were excluded.

### Inclusion and exclusion criteria

Patients were included if they met the following criteria: (1) histologically or cytologically confirmed pancreatic cancer; (2) deemed unresectable by a multidisciplinary team based on imaging and clinical evaluation; (3) received chemoradiotherapy as primary treatment; and (4) had complete baseline clinical and survival information.

Exclusion criteria were: (1) prior surgical resection of the primary tumor; (2) presence of another primary malignancy; (3) incomplete treatment or loss to follow-up within 1 month; (4) missing key clinical variables or survival data.

### Ethical considerations

All patients provided written informed consent prior to receiving chemoradiotherapy. This study was conducted in accordance with the principles of the Declaration of Helsinki and relevant national guidelines. Given the retrospective nature of the research and the complete anonymization of patient data, the Ethics Committee of Henan Provincial People’s Hospital waived the requirement for additional informed consent and formal ethical approval.

### Data

Clinical and laboratory data were collected from institutional databases. No missing data were present among the variables used for model construction, as patients with incomplete clinical or laboratory parameters were excluded during initial screening. At baseline, the following variables were collected for each patient: demographic characteristics, including age (reported as mean ± standard deviation) and sex (male or female); clinical staging information, including overall stage (I–IV), T stage (1–4 or x), N stage (0–2 or x), and M stage (M0 or M1), all classified according to the American Joint Committee on Cancer (AJCC) 8th edition criteria; tumor-related features such as maximum tumor diameter (in centimeters); hematologic markers including white blood cell count (WBC, ×10^9^/L), lymphocyte count (×10^9^/L), and platelet count (PLT, ×10^9^/L); and serum tumor marker levels, specifically carbohydrate antigen 19-9 (CA19-9, U/mL). Overall survival (OS) was defined as the time from the initiation of chemoradiotherapy to death from any cause or last follow-up, whichever occurred first.

### AI-model construction and evaluation

All 214 patients were randomly divided into a training cohort and a validation cohort at a ratio of 6:4. In the training cohort, five machine learning–based survival models were developed to predict OS: LASSO, Cox, RSF, SVM, and XGBoost. The concordance index (C-index) was calculated to assess the discrimination performance of each model in the training cohort.

In the validation cohort, time-dependent receiver operating characteristic (ROC) curves, decision curve analysis (DCA), and calibration curves were used to evaluate the predictive accuracy, clinical net benefit, and calibration performance of each model, respectively. To enhance model interpretability, feature importance rankings and SHapley Additive exPlanations (SHAP) values were generated for AI-model in the training cohort.

### Statistical analysis

Group comparisons for categorical variables were performed using the chi-square test or Fisher’s exact test, depending on sample size and distribution. For continuous variables, the Student’s t-test was used for normally distributed data, while the Mann–Whitney U test was applied when the normality assumption was not met. OS was analyzed with the Kaplan–Meier approach, and survival differences between subgroups were examined using the log-rank test. All artificial intelligence models were implemented using R software. A p-value less than 0.05 (two-tailed) was considered indicative of statistical significance.

## Result

### Patients

A total of 214 patients with unresectable pancreatic cancer were included in the study. The mean age was 61.4 years (SD = 10.5), and 56.1% were male. Most patients presented with advanced disease: 55.6% were at stage IV, 25.2% at stage III, and only 8.88% and 10.3% at stages I and II, respectively. For T stage, the majority were classified as T4 (44.4%), while T1, T2, and T3 accounted for 3.74%, 20.6%, and 17.3%, respectively. In terms of nodal involvement, 43.9% were N0, 33.2% N1, and 7.48% N2, with 15.4% having unknown N stage. More than half (55.6%) of the cohort had distant metastases (M1).

The cohort was randomly divided into a training set (n = 127) and a validation set (n = 87). Baseline characteristics were generally balanced between the two groups; however, a statistically significant difference was observed in clinical stage distribution (p = 0.028, **Table 1**).

**Table 1:** Baseline characteristics of the whole cohort, training set, and validation set.

Variable	Total (N = 214)	Validation set (N = 87)	Training set (N = 127)	P-value
Age, mean (SD)	61.4 (10.5)	61.6 (9.88)	61.3 (10.9)	0.825
Sex, n (%)				0.719
Female	94 (43.9)	40 (46.0)	54 (42.5)	
Male	120 (56.1)	47 (54.0)	73 (57.5)	
Stage, n (%)				0.028*
I	19 (8.88)	4 (4.60)	15 (11.8)	
II	22 (10.3)	13 (14.9)	9 (7.09)	
III	54 (25.2)	27 (31.0)	27 (21.3)	
IV	119 (55.6)	43 (49.4)	76 (59.8)	
T stage, n (%)				0.427
1	8 (3.74)	2 (2.30)	6 (4.72)	
2	44 (20.6)	15 (17.2)	29 (22.8)	
3	37 (17.3)	15 (17.2)	22 (17.3)	
4	95 (44.4)	45 (51.7)	50 (39.4)	
x	30 (14.0)	10 (11.5)	20 (15.7)	
N stage, n (%)				0.831
0	94 (43.9)	38 (43.7)	56 (44.1)	
1	71 (33.2)	31 (35.6)	40 (31.5)	
2	16 (7.48)	5 (5.75)	11 (8.66)	
x	33 (15.4)	13 (14.9)	20 (15.7)	
M stage, n (%)				0.172
M0	95 (44.4)	44 (50.6)	51 (40.2)	
M1	119 (55.6)	43 (49.4)	76 (59.8)	
Tumor size, cm	4.27 (1.83)	4.17 (1.77)	4.34 (1.87)	0.518
WBC, ×10^9^/L	6.35 (3.62)	5.90 (2.44)	6.65 (4.23)	0.102
Lymphocytes, ×10^9^/L	1.46 (0.74)	1.46 (0.59)	1.45 (0.83)	0.92
PLT, ×10^9^/L	234 (102)	230 (92.4)	237 (108)	0.609
CA199, U/mL	1219 (2528)	1044 (2286)	1339 (2684)	0.39

*Statistically significant (p < 0.05).

WBC, white blood cell count; PLT, platelet count; CA199, carbohydrate antigen 19-9.

### OS

**Figure 1:**
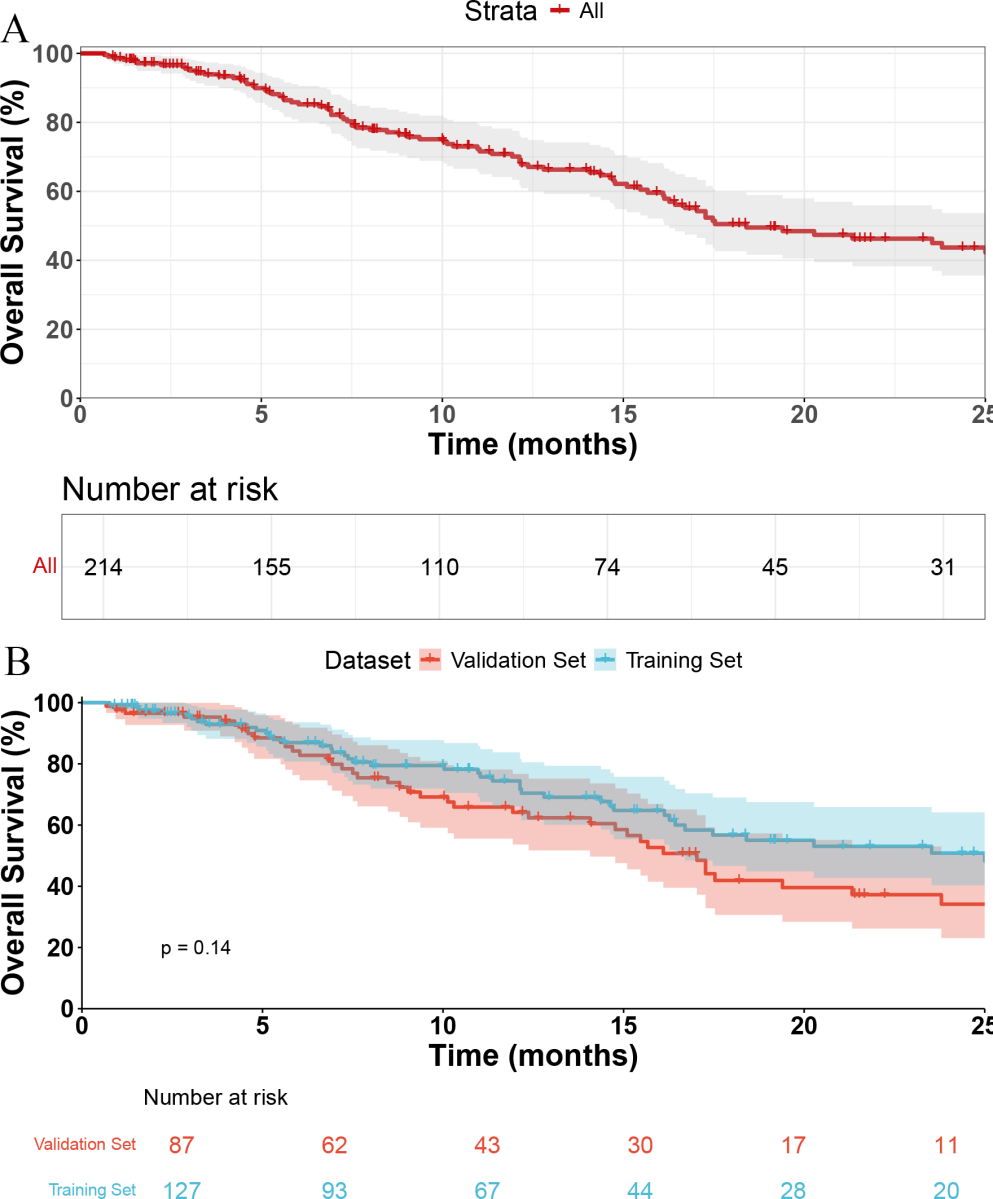
Kaplan-Meier curves of overall survival. **(A)** Overall survival of all patients. **(B)** Comparison of overall survival between the training set and validation set.

The median OS (mOS) for the entire cohort was 18.4 months (95% confidence interval [CI], 16.3–28.1, **Figure 1A**). No significant difference in mOS was observed between the training and validation cohorts, with median survival times of 25 months and 17 months, respectively (p = 0.14, **Figure 1B**).

### AI-model

**Figure 2:**
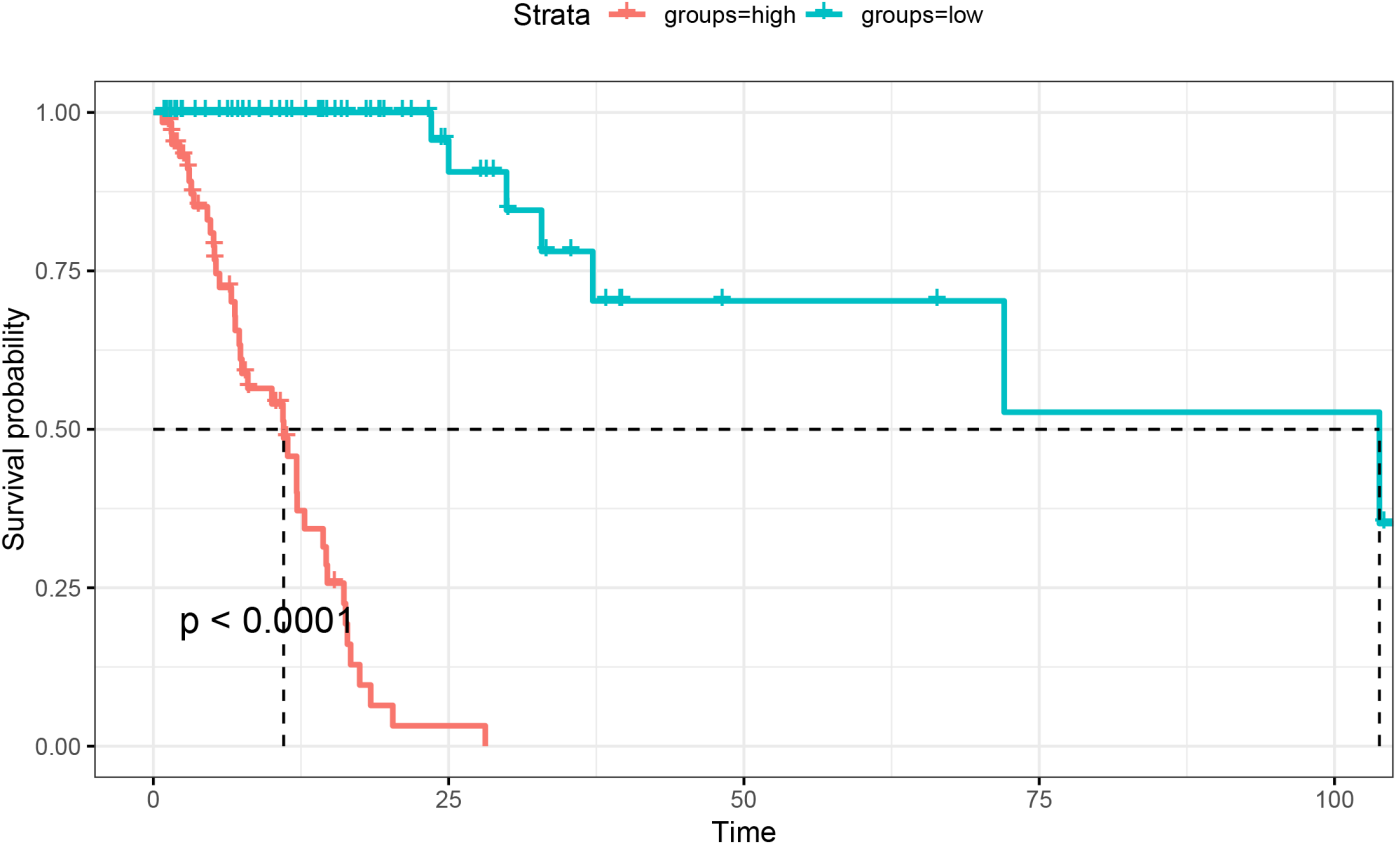
Kaplan-Meier survival curves based on XGBoost-predicted risk groups in the training set.

In the training cohort, five models were constructed to predict 0S. The concordance indices (C-index) were 0.716 for LASSO, 0.740 for Cox, 0.940 for RSF, 0.712 for SVM, and 0.949 for XGBoost. XGBoost not only demonstrated the highest discriminative ability among the five models, with a C-index of 0.949, but also enabled calculation of individualized risk scores. Patients classified into the high-risk group by the XGBoost model showed significantly worse overall survival compared to the low-risk group (p < 0.001, **Figure 2**).

**Figure 3:**
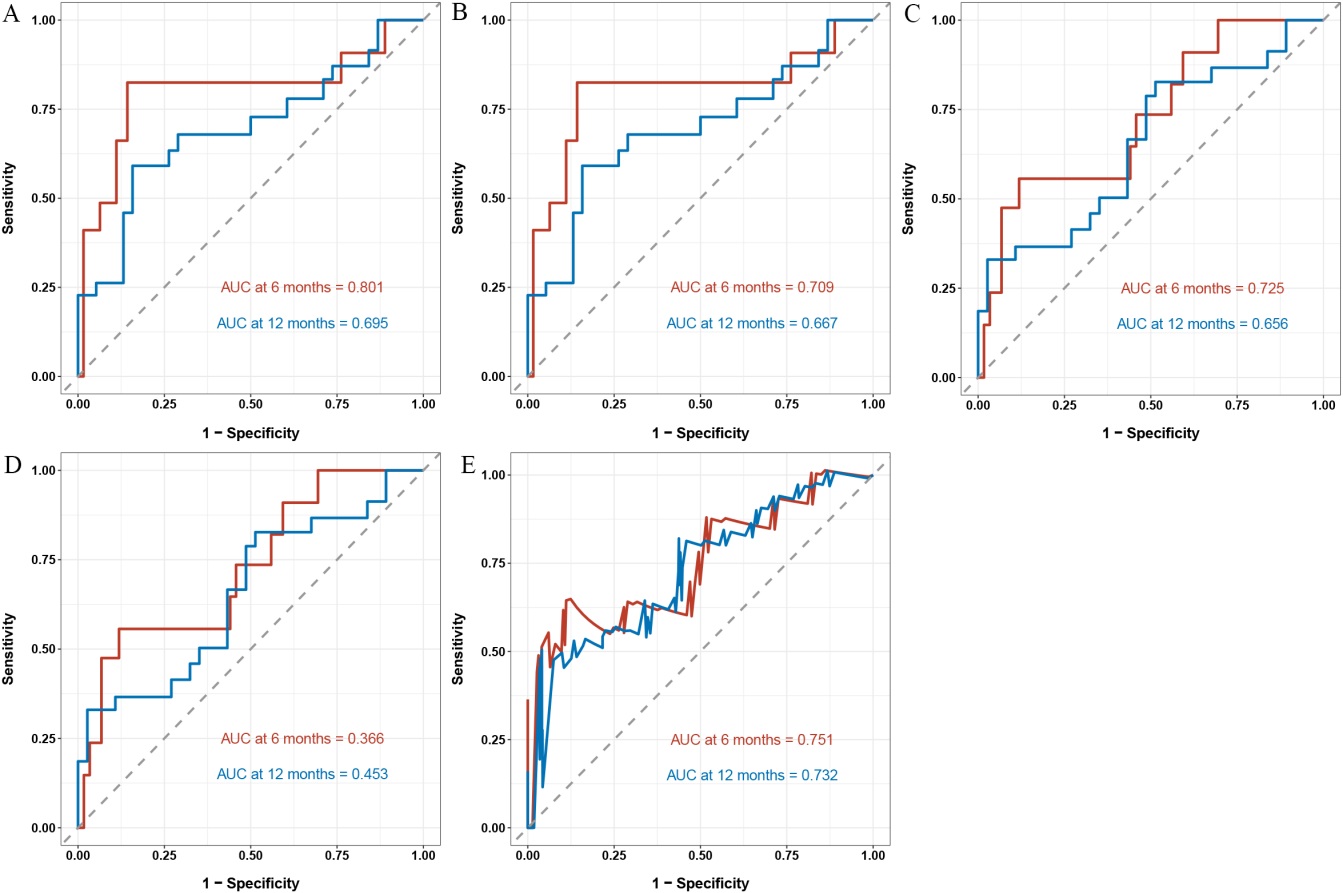
Time-dependent ROC curves for survival prediction at 6 and 12 months in the validation cohort. Cox **(A)**, LASSO **(B)**, RSF **(C)**, SVM **(D)**, and XGBoost **(E)**.

**Figure 4:**
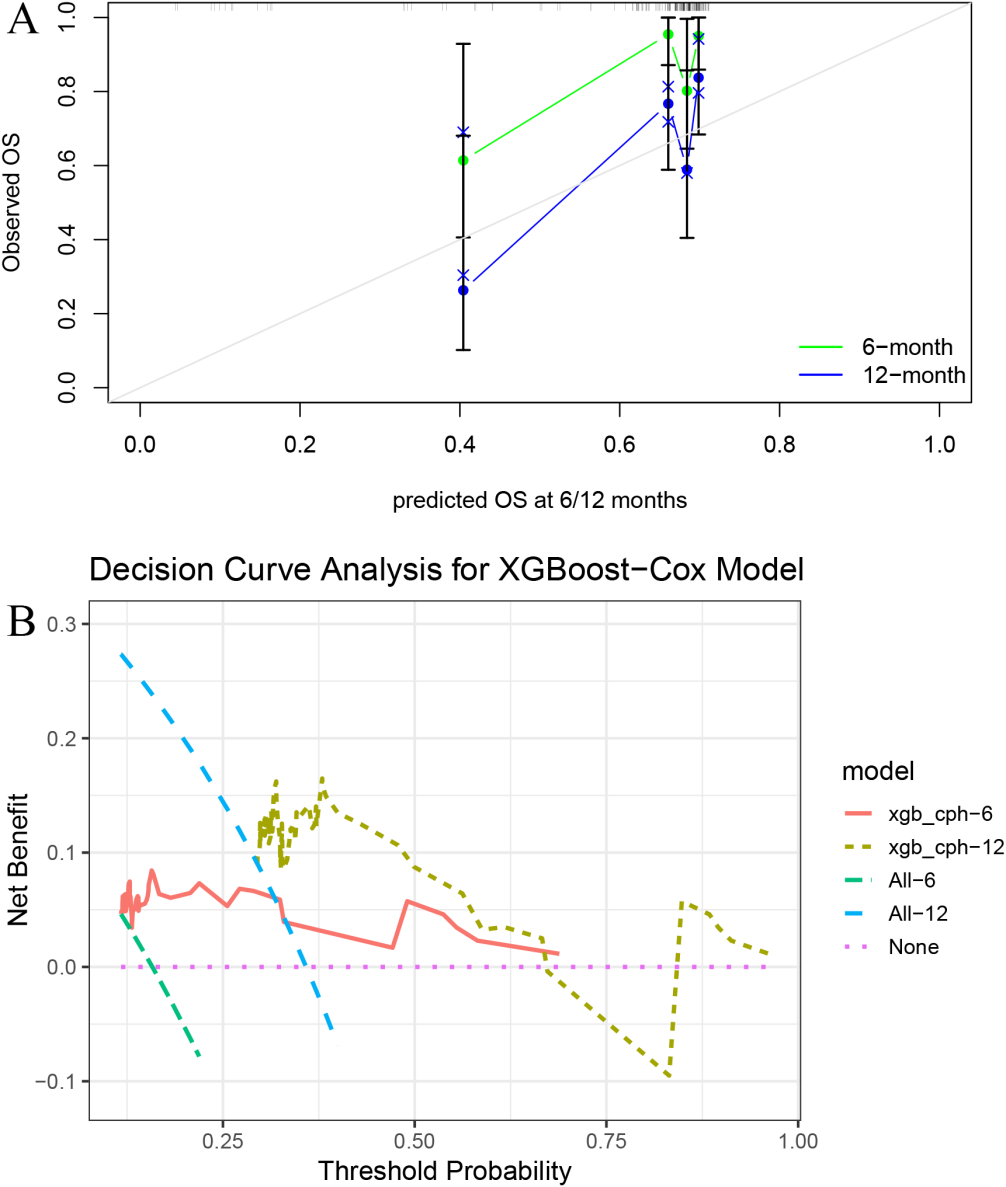
Calibration and decision curve analysis for the XGBoost model. **(A)** Calibration plots at 6 and 12 months show good agreement between predicted and observed survival. **(B)** Decision curve analysis (DCA) demonstrates the net clinical benefit of the model across a range of threshold probabilities.

In the validation cohort, the time-dependent area under the ROC curve (AUC) at 6 and 12 months was as follows: for Cox (**Figure 3A**), 0.801 and 0.692; for LASSO (Figure3B), 0.709 and 0.667; for RSF (**Figure 3C**), 0.725 and 0.656; for SVM (**Figure 3D**), 0.366 and 0.453; and for XGBoost (**Figure 3E**), 0.751 and 0.732. The calibration plots (**Figure 4A**) showed close alignment between predicted and actual survival outcomes for the XGBoost model at both 6- and 12-month time points, suggesting strong consistency in its risk estimation. Additionally, DCA (**Figure 4B**) revealed that XGBoost provided a higher net clinical benefit than the other models across a wide range of threshold probabilities.

**Figure 5:**
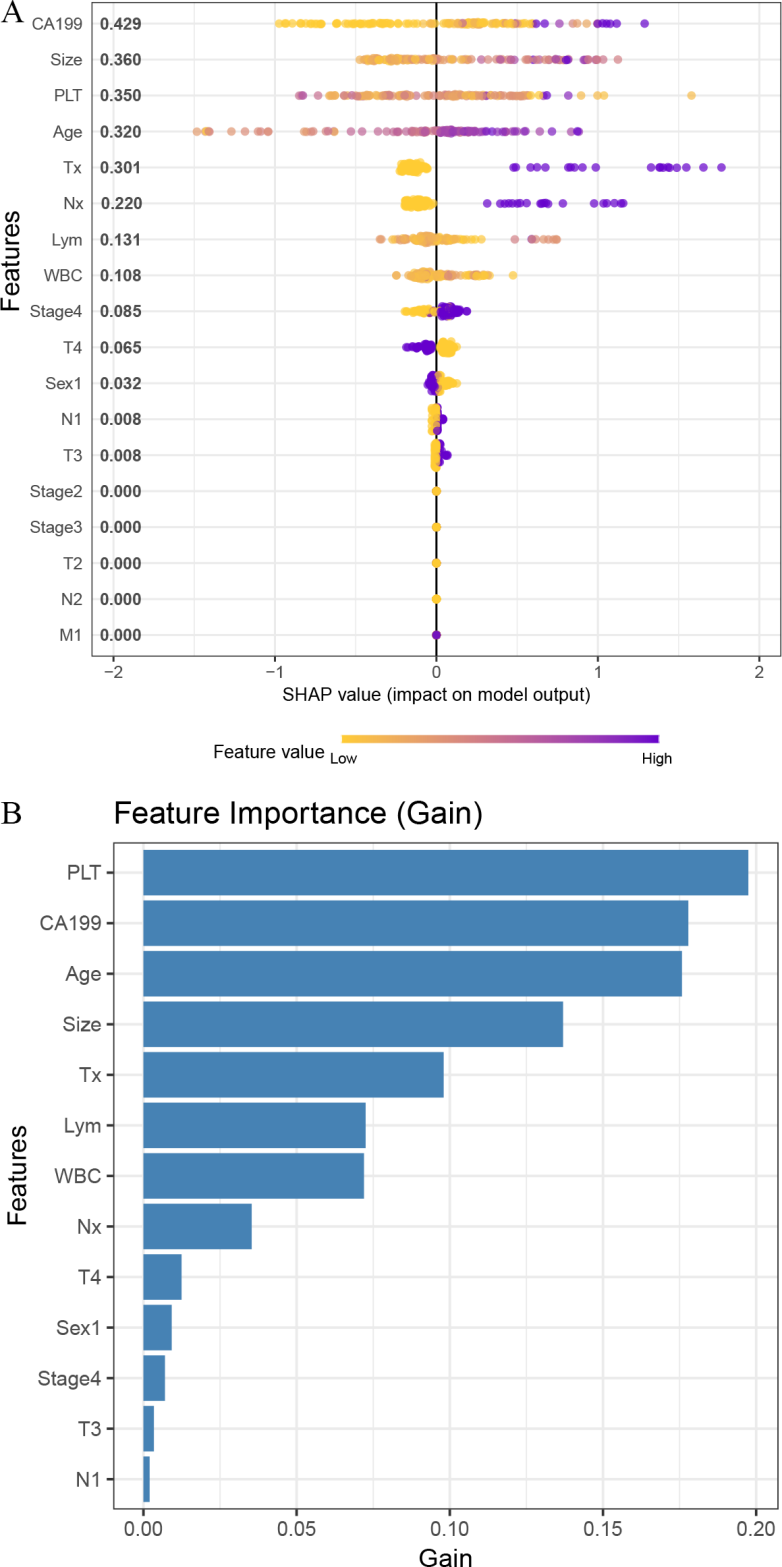
Feature importance analysis of the XGBoost model. **(A)** SHAP summary plot showing the impact of each feature on model output. **(B)** Bar plot of feature importance ranked by gain in the XGBoost model.

**Figure 5A** shows that CA199, tumor size, PLT, and age had the greatest impact on the model output based on SHAP values, while **Figure 5B** indicates that PLT, CA199, and age were the top contributors to the model based on Gain importance.

## Discussion

CRT has become a cornerstone in the treatment of patients with unresectable pancreatic cancer, offering improved local control and a chance of survival extension (14). However, prognosis remains highly variable and difficult to predict using conventional indicators. While staging systems and biomarkers like CA199 are commonly applied, they lack the ability to fully capture patient heterogeneity (15, 16). Moreover, only a limited number of studies have explored predictive modeling specifically in this population, and even fewer have applied AI techniques to enhance individualized risk assessment.

CRT remains particularly important in cases where surgery is not feasible due to vascular involvement, distant metastasis, or medical comorbidities. It can provide meaningful symptom relief and disease stabilization (17, 18). Nonetheless, clinical outcomes vary widely. Some patients derive substantial benefit, while others experience rapid progression. This discrepancy underscores the need for more advanced models that can incorporate complex variable interactions and provide personalized survival predictions beyond conventional methods.

In this study, we compared five modeling strategies—Cox, LASSO, RSF, SVM, and XGBoost—to identify an optimal survival prediction framework for CRT-treated patients. XGBoost exhibited the highest predictive performance, achieving a C-index of 0.949 in the training set, and outperformed the other models in time-dependent AUCs at 6 and 12 months. Calibration and decision curve analyses confirmed the consistency and net clinical benefit of its predictions. This superior performance is likely due to the algorithm’s ability to model non-linear relationships, account for complex variable interactions, and reduce overfitting through regularization. Additionally, its capacity to manage missing or noisy data makes it well-suited for real-world clinical datasets (19–22).

To address the interpretability challenge often associated with AI models, we utilized SHAP to evaluate the relative importance of input variables. As shown in **Figure 5A**, CA19-9, tumor size, PLT, and age emerged as the most impactful predictors. These findings are consistent with established clinical and biological knowledge. CA19–9 is a widely used tumor marker in pancreatic cancer and serves as a surrogate for tumor burden. Elevated CA19–9 levels are associated with more aggressive tumor biology, advanced disease stage, and poor prognosis, and are routinely used to monitor treatment response and recurrence. Tumor size is directly related to tumor invasiveness and treatment response, with larger tumors often indicating a higher likelihood of vascular invasion, local progression, and reduced effectiveness of chemoradiotherapy. Elevated platelet counts may reflect a tumor-driven pro-thrombotic and inflammatory microenvironment, which can promote cancer progression, angiogenesis, and immune evasion. Age is a fundamental prognostic factor across malignancies, as older patients often have decreased physiological reserves, reduced immune function, and increased comorbidity burdens, all of which may impact treatment tolerance and overall survival. The identification of these readily accessible variables not only supports the clinical relevance of the model but also reinforces its potential for real-world application in guiding personalized treatment planning (23–26).

The model developed in this study offers a clinically practical and easily implementable approach for stratifying survival risk in patients with unresectable pancreatic cancer—a population with limited treatment options. It relies on routinely available clinical and laboratory variables, including CA19-9, platelet count, tumor size, and patient age, all of which are typically collected during the initial evaluation. This makes integration into standard clinical workflows highly feasible. By inputting these parameters into a web-based risk calculator, clinicians could classify patients into distinct risk categories and tailor management strategies accordingly. High-risk patients could be prioritized for intensified chemoradiotherapy regimens, more frequent surveillance imaging, or early referral to clinical trials, whereas low-risk patients might be managed with standard protocols and spared from overtreatment. Importantly, the use of SHAP values enhances model transparency by identifying key features driving individual predictions, thereby improving interpretability and fostering clinical trust. Future efforts will focus on developing an accessible online tool to facilitate real-time, individualized decision support based on this model (27).

Despite its strengths, the study has limitations. As a retrospective analysis, there is a risk of selection bias and unmeasured confounding. Although patient eligibility was carefully defined and data completeness was ensured, prospective validation is necessary. The sample size, while sufficient for initial modeling, may limit the generalizability of findings. Validation in larger, multi-institutional cohorts would be an important next step. Furthermore, the model was built using clinical and laboratory variables only. Incorporating imaging-derived features or molecular data could potentially enhance predictive accuracy and offer mechanistic insights into disease progression. In addition, while SHAP improves interpretability, tree-based models such as XGBoost still require cautious application when used in clinical decision-making. Finally, although the XGBoost model demonstrated excellent predictive performance, the possibility of model overfitting cannot be completely ruled out. Our current validation approach was based on an internal split of the multicenter dataset, and no external cohort was used. Therefore, external validation in larger, geographically distinct populations is necessary to confirm the model’s generalizability and clinical utility. Additionally, future work may benefit from implementing k-fold cross-validation or nested cross-validation strategies to further minimize overfitting risk and enhance robustness.

## Conclusion

In conclusion, we successfully constructed an AI-based model using XGBoost to predict survival in patients with unresectable pancreatic cancer undergoing CRT. CA199, tumor size, PLT, and age were identified as the most important prognostic factors. The model demonstrated high accuracy, strong calibration, and clinical utility, offering a promising tool for individualized risk estimation and treatment guidance. Further validation with larger datasets and integration of additional data modalities will be essential to enhance the robustness and applicability of this approach.

## Data Availability

The original contributions presented in the study are included in the article/supplementary material. Further inquiries can be directed to the corresponding author.
